# Non-alcoholic fatty liver disease does not increase dementia risk although histology data might improve risk prediction

**DOI:** 10.1016/j.jhepr.2020.100218

**Published:** 2020-12-01

**Authors:** Ying Shang, Patrik Nasr, Mattias Ekstedt, Linnea Widman, Per Stål, Rolf Hultcrantz, Stergios Kechagias, Hannes Hagström

**Affiliations:** 1 Department of Neurobiology, Care Sciences and Society (NVS), Karolinska Institutet, Stockholm, Sweden; 2 Department of Gastroenterology and Hepatology, Department of Health, Medicine and Caring Sciences, Linköping University, Linköping, Sweden; 3 Division of Biostatistics, Institute of Environmental Medicine, Karolinska Institutet, Stockholm, Sweden; 4 Division of Hepatology, Department of Upper GI, Karolinska University Hospital, Stockholm, Sweden; 5 Department of Medicine, Huddinge, Karolinska Institutet, Stockholm, Sweden; 6 Clinical Epidemiology Unit, Department of Medicine, Stockholm, Karolinska Institutet, Stockholm, Sweden

**Keywords:** NAFLD, Histological pathology, Dementia, Prediction

## Abstract

**Background & Aims:**

Non-alcoholic fatty liver disease (NAFLD) is common in the general population, but its association with dementia is unclear. We aimed to assess the risk of dementia related to NAFLD, and to determine whether histological parameters could improve the predictive capacity of a conventional risk model for dementia in patients with biopsy-proven NAFLD.

**Methods:**

A retrospective matched cohort study of 656 NAFLD patients underwent liver biopsy at 2 hospitals between 1971 and 2009. Up to 10 individuals (controls) from the general population (n = 6,436) were matched for age, sex, and municipality to each patient. Dementia was ascertained from National registers until 2014. Using Cox regression, we estimated hazard ratios for dementia with 95% confidence intervals. In the biopsy cohort, the discriminative power of adding histological markers to a conventional risk model was assessed by Harrell’s C-index and compared with a likelihood-ratio test.

**Results:**

During a mean follow-up of 19.7 ± 8.7 years, 3.3% of the NAFLD patients and 4.9% of the controls developed dementia (*p* = 0.07). Overall, NAFLD was not significantly associated with incident dementia. In the biopsy cohort, the model of conventional risk factors (age, sex, hypertension, and cardiovascular diseases) had a C-index of 0.912 to predict incident dementia. Adding individual histological parameters significantly increased the prediction of dementia, with the most pronounced improvement for fibrosis stage (C-index = 0.938, *p* <0.05).

**Conclusions:**

Although NAFLD was not associated with the risk of dementia, we found that adding histological markers to a conventional risk model for dementia enhanced the predictive capacity, indicating a shared metabolic origin.

**Lay summary:**

Both non-alcoholic fatty liver disease (NAFLD) and dementia are increasing in prevalence because of a more sedentary lifestyle, increased prevalence of obesity and population ageing. However, the link between these 2 diseases is not well studied. We investigated the association between NAFLD and the risk of dementia and found no association. However, liver histology parameters, especially fibrosis, could significantly improve the prediction of dementia risk.

## Highlights

• The link between NAFLD and dementia is unclear.
• We assessed the association between biopsy-proven NAFLD and dementia.
• NAFLD is not associated with an increased risk of dementia.
• Adding histological markers to a conventional risk model for dementia enhanced its predictive capacity.

## Graphical abstract

**Figure:**
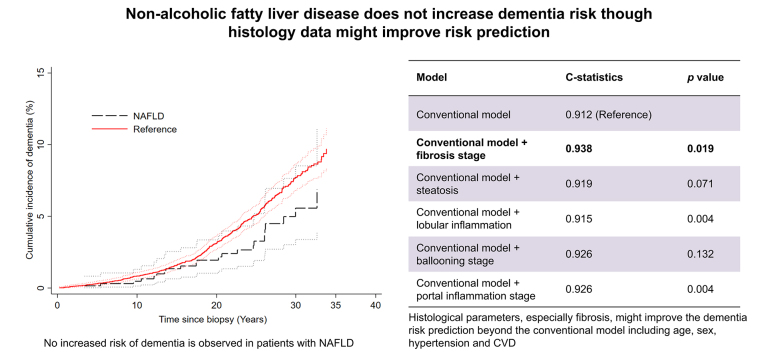

## Introduction

Non-alcoholic fatty liver disease (NAFLD) is a common cause of chronic liver disease, with a prevalence of 25% in the general population and >80% in the morbidly obese population.^1^^,^^2^ Accumulating evidence suggests that NAFLD is associated with cardiovascular diseases (CVDs).^3^ It has also been postulated that NAFLD may be linked to an increased risk for dementia, as these conditions share common risk factors (*e.g.* older age, type 2 diabetes mellitus [T2DM] and hypertension)^4^ and possibly common pathophysiological mechanisms (*e.g.* oxidative stress and inflammation).^5^ Indeed, several studies have shown that NAFLD is associated with both poorer cognitive performance,[6], [7], [8] and low total cerebral brain volume independently of cardiometabolic disorders.^4^ However, these studies were performed in a cross-sectional fashion and none have examined its association with incident dementia − a typical clinical manifestation associated with ageing. Given the ageing population and the high prevalence of dementia and NAFLD, it is essential to understand whether NAFLD increases the risk of dementia. However, to our knowledge the longitudinal association has not yet been widely investigated.

NAFLD can be defined and graded by liver biopsy, which is the gold standard for both the definitive diagnosis and a severity assessment of NAFLD.^9^ Although advanced liver diseases are often accompanied by cognitive deficits (*i.e.* hepatic encephalopathy), a recent Framingham study added a piece of evidence that, within NAFLD patients, advanced fibrosis is independently associated with cognitive impairment.^10^ This study, however, is also cross-sectional and the cases were ascertained using the NAFLD fibrosis score, which has a low sensitivity (between 50 and 67%) for advanced fibrosis.^11^ Furthermore, because the metabolic environment is altered in NAFLD patients,^12^ it is uncertain whether established risk factors (such as CVDs) are still associated with incident dementia in patients with NAFLD and whether adding histological parameters improves predictive capacity beyond these risk factors. This might be of clinical relevance as previous models for predicting risk of dementia mainly included sociodemographic and cardiometabolic risk factors for the general population or patients with diabetes,^13^ and a specific dementia risk model for patients with NAFLD have not been presented. Therefore, this study aims to: (1) investigate the risk of incident dementia in patients with NAFLD compared with matched reference individuals from the general population (controls); (2) examine the risk of dementia related to different characteristics and severity of NAFLD within the NAFLD patients; and (3) evaluate whether adding histological parameters could improve the predictive capacity of dementia over conventional risk factors.

## Methods and materials

### Settings and data sources

**Fig. 1:**
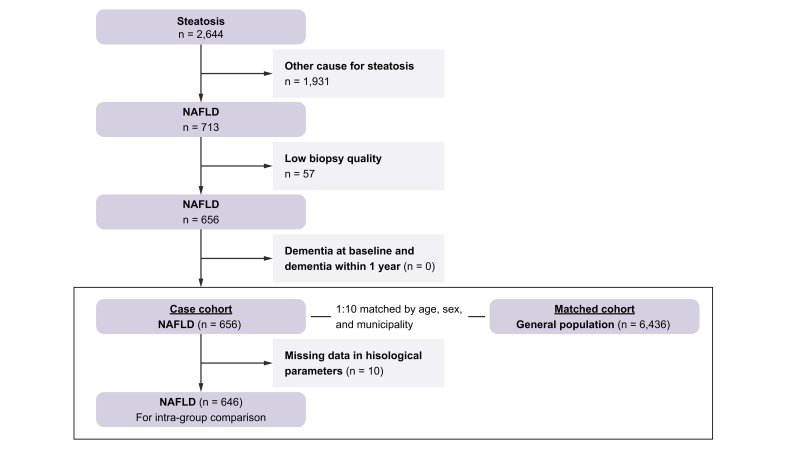
Flowchart of the study population. NAFLD, non-alcoholic fatty liver disease.

We conducted a retrospective matched cohort study comprising all patients diagnosed with biopsy-proven NAFLD from Karolinska University Hospital, Huddinge and Linköping University Hospital from January 1971 to December 2009. A detailed description of identifying NAFLD patients has been published elsewhere.^14^ Briefly, all biopsies were categorised by a pathologist at the time of biopsy based on the systematised nomenclature of medicine (SNOMED).^15^ The code for hepatic steatosis (M50080) was used to identify all liver biopsies with steatosis (n = 2,644). The medical charts of all patients were scrutinised. We excluded patients with the following conditions: having causes for steatosis other than NAFLD or diagnosed with concurrent liver disease at baseline or during follow-up; reporting daily alcohol consumption of more than 30 g for men or 20 g for women at baseline or during follow-up; and reporting on treatment with drugs associated with steatosis or hepatotoxicity at the time of biopsy. In addition, we excluded patients with a diagnosis of dementia at baseline and within 1 year of follow-up (Fig. 1).

### Histopathological evaluation

All liver biopsies were well archived and preserved after the initial assessment. In the slides with faded staining, new sections and staining were performed. We excluded 57 biopsies showing the absence of steatosis or insufficient quality (*e.g.* macerated during the staining process) or size (<7 portal tracts). One expert liver pathologist (RH) reviewed all of the available biopsies, which were scored based on the NAFLD activity score (NAS).^9^ Notably, 69 liver biopsies had been previously reassessed by another experienced pathologist as part of a previous follow-up study.^16^ These 69 liver biopsies were used for fibrosis staging but were not available for reassessment for analysis of activity scores because of low reproducibility for lobular inflammation and ballooning between the two pathologists (Cohen’s kappa = 0.062). However, they were still included in the analyses of fibrosis stage, as the agreement on fibrosis stage between the two pathologists was substantially higher (Cohen’s kappa =  0.73). The fatty liver inhibition of the progression algorithm was used to define the presence of non-alcoholic steatohepatitis (NASH).^17^^,^^18^ Lobular inflammation and steatosis were scored on a 4-point (0–3) scale and ballooning and portal inflammation on a 3-point (0–2) scale.^9^ Fibrosis stage was scored on a 5-point (0–4) scale.^9^

### Baseline characteristics

Health conditions and diagnoses at the time of liver biopsy were registered from patient charts, as described previously.^14^ Hypertension was defined as a registered diagnosis from the patient chart, a resting blood pressure of ≥140/90 mmHg or having any antihypertensive medication. T2DM was defined as a registered diagnosis from the patient chart, non-fasting glucose of ≥180 mg/dl or a fasting glucose of ≥126 mg/dl or having any antidiabetic medication prescribed. Hyperlipidaemia was defined as a fasting total cholesterol level of ≥240 mg/dl or having been prescribed any antilipidemic drug. CVDs were defined as the first event of any acute ischaemic heart disease, ischaemic stroke or haemorrhagic stroke,^3^ and were identified from the National Patient Register of Hospital Discharges (NPR). Smoking status was categorised as ever *vs.* never smoking. Weight and height were measured objectively by hospital staff and BMI was calculated as weight (kg) divided by the square of height (m). Obesity was further defined as BMI over 30 kg/m^2^.

### Biochemical variables

Information on alanine aminotransferase (ALT) and aspartate aminotransferase (AST), albumin, bilirubin, alkaline phosphatase (ALP), gamma-glutamyl transferase (GGT) levels, complete blood count, fasting cholesterol and triglycerides, fasting glucose, autoantibodies and alpha1-antitrypsin levels were registered and collected from routine biochemical variables within 1 month of liver biopsy. Multiple imputations were applied to address missing data.

### Matched comparison cohort

All Swedish permanent residents are assigned a unique 10-digit personal identification number.^19^ Each patient in the histological cohort was matched for sex, age, and municipality at the time of biopsy with up to 10 randomly selected individuals from the general population (controls) derived from Statistics Sweden. We excluded control individuals with a pre-existing diagnosis of dementia at the time of matching.

### Follow-up and outcomes

In total of 656 NAFLD patients and 6,436 controls were cross-linked to the NPR and the Cause of Death Register (CDR). The validity of the NPR is generally considered high, with a previous study confirming a positive predictive value between 85 and 95% for different diseases.^20^ The CDR includes information on the date and cause of death for all Swedish inhabitants (who died in or outside Sweden), with completeness exceeding 99%.^21^ All cases of dementia and its subtype were identified from the registries according to codes from the International Classification of Disease (ICD) versions 8–10. Dementia was further classified into 2 major subtypes−Alzheimer’s disease (AD) and non-Alzheimer’s disease dementia (non-AD). CVDs during follow-up were also identified for both NAFLD patients and matched controls.^3^ All diagnosis codes to define dementia and its subtype and CVDs are presented in Table S1. Both NAFLD patients and controls were followed up from the index date to the date of dementia identification, death, or December 31, 2014, whichever occurred first.

### Statistical analysis

Descriptive statistics were calculated for available variables in NAFLD patients, with continuous variables expressed as mean and standard deviations and categorical variables as total number and percentages. Two main analyses were performed. First, we tested whether the risk of dementia was increased in the NAFLD cohort *vs.* the reference population. Here, we adjusted for matching variables (age, sex, municipality) and ICD-based diagnoses (CVDs) as possible confounders. More detailed data, such as lab parameters, were not available in the reference population. Of 656 patients with NAFLD, 10 persons had liver-related events within 6 months of baseline and were excluded from the histopathological evaluation; however, they were included here to ascertain dementia risk. The incidence rates of dementia and its subtypes in NAFLD patients and matched controls were calculated as the number of cases divided by the total person-time accrued during follow-up. The cumulative incidence of dementia was calculated for NAFLD patients and their matched controls separately using a competing risk regression model with overall mortality as the competing event. We estimated hazard ratios (HRs) and 95% confidence intervals (CIs) for dementia in patients with NAFLD compared with the matched controls from stratified Cox proportional hazard regression models using time since index date as the timescale. The association between NAFLD and dementia risk was first estimated conditionally on only matching variables and then additionally adjusted for ICD-codes representative of CVD as time-varying covariates. The proportional hazard assumption for the Cox models was tested and fulfilled using Schoenfeld residuals.

Second, to investigate whether adding histological parameters increases the predictability of dementia risk we restricted our analysis to the NAFLD cohort with more detailed data. First, we performed several individual Cox regression models to identify the risk factors that most strongly associated with dementia risk in the histological cohort. Previous literature suggests that age, female sex, T2DM, CVDs, hypertension, smoking, and obesity are common risk factors for dementia.^22^ We then tested the association between these risk factors and the risk of dementia by entering them one by one into a univariate Cox model; age, female sex, hypertension, and CVDs were the factors with *p* <0.1 in the univariate Cox model and therefore these factors were entered into a multi-adjusted Cox regression model, serving as a baseline model. We excluded T2DM, smoking, and obesity in the final model seeing that these risk factors were not associated with dementia in this cohort.

Harrell’s C-index was used to investigate the predictive ability of the models. We used a likelihood-ratio (LR) test to examine whether adding histological parameters significantly improved the predictive power of dementia. All analyses were performed using STATA 15.0 software (StataCorp, College Station, TX, USA).

### Sensitivity analysis

We performed several sensitivity analyses to examine the robustness of our results: (1) we first excluded NAFLD without histological information (n = 10) in the analysis of the association between NAFLD and dementia relative to the controls; (2) we excluded study participants <35 years of age at baseline (n = 1225), as these would have a low risk for incident dementia within the possible maximal follow-up period; and (3) within the NAFLD cohort, we added smoking, T2DM, and obesity to the baseline model to investigate whether adding histological parameters could still improve the predictive ability for dementia over the model including all the common risk factors available in the dataset.

### Ethical consideration

The study was approved by the regional ethics committee at Karolinska Institutet and Linköping University (Dnr 2011/905-31/2 and 2015/1591-32). Informed consent was waived by the ethics committee because of the anonymisation of patient data.

## Results

In total, 656 patients with NAFLD and 6,436 comparison individuals matched on age, sex, and municipality were included in the study population. The mean ages were 48.2 (±SD 13.7) years in NAFLD and 48.4 (±SD 13.6) in the matched cohort. More than half (62.2%) of the patients were men. Median follow-up was 20.1 years (inter-quartile range [IQR] 1.4–35.9) in the patient cohort and 20.5 (IQR 0.1–40.0) in the matched cohort. Of the patients with NAFLD, 646 had information on histological parameters. The baseline characteristics of the histological cohort are listed in Table 1. There were 196 (30.3%) patients with hypertension and 1 (1.3%) with a history of CVDs.

**Table 1:** Baseline demographic, clinical, and histopathological characteristics of the study population in biopsy-proven NAFLD (n = 646).

Parameters	Complete data, N	Mean/frequency (±SD/%)
NASH	577	383 (66.4)
Fibrosis stage 0	646	164 (25.4)
Fibrosis stage 1		256 (23.6)
Fibrosis stage 2		149 (23.1)
Fibrosis stage 3		58 (9.0)
Fibrosis stage 4		20 (3.1)
Steatosis grade 0	580	0 (0)
Steatosis grade 1		228 (39.3)
Steatosis grade 2		149 (25.7)
Steatosis grade 3		203 (35.0)
Lobular inflammation 0	579	52 (9.0)
Lobular inflammation 1		245 (42.3)
Lobular inflammation 2		220 (38.0)
Lobular inflammation 3		62 (10.7)
Ballooning 0	577	189 (32.8)
Ballooning 1		207 (35.9)
Ballooning 2		181 (31.4)
Portal inflammation 0	579	254 (43.9)
Portal inflammation 1		226 (39.0)
Portal inflammation 2		99 (17.1)
Age at biopsy (years)	646	48.2 (±13.7)
Sex (male)	646	402 (62.2)
Type 2 diabetes	646	93 (14.4)
Hypertension	646	196 (30.3)
Hyperlipidaemia	646	57 (8.8)
Smoking (never)	569	355 (55.0)
Smoking (ever)		291(45.0)
BMI (kg/m^2^)	546	28.3 (±4.1)
Cardiovascular disease	646	12 (1.3)
Haemoglobin (g/dl)	561	14.8 (±1.2)
AST (U/L)	631	50 (±34)
ALT (U/L)	632	84 (±52)
GGT (U/L)	540	109 (±127)
ALP (U/L)	625	91 (±47)
Albumin (g/L)	573	4.2 (±0.4)
Ferritin (μg/L)	355	237 (±249)
Total cholesterol (mg/dl)	462	233 (±54)
Triglycerides (mg/dl)	430	208 (±146)
Fasting glucose (mg/dl)	430	108 (±40)

ALP, alkaline phosphatase; ALT, alanine transaminase; AST, aspartate transaminase; GGT, gamma-glutamyltransferase; NAFLD, non-alcoholic fatty liver disease; NASH, non-alcoholic steatohepatitis.

### Incident dementia in NAFLD patients vs. controls

**Fig. 2:**
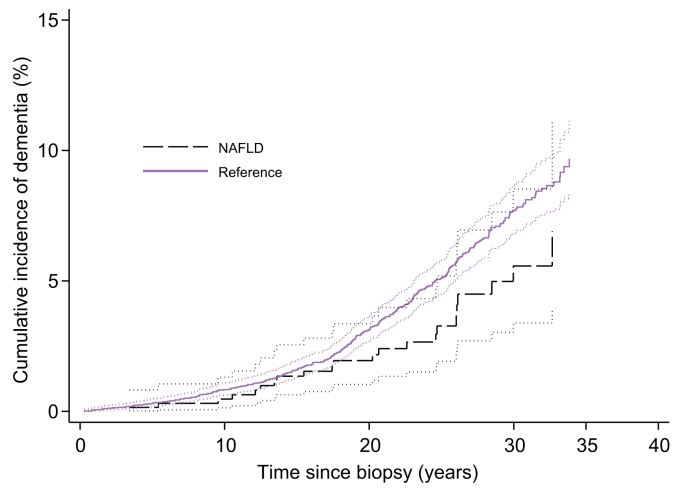
Cumulative incidence of dementia among NAFLD patients and their matched reference individuals from the general population. NAFLD, non-alcoholic fatty liver disease.

During the follow-up (mean 19.6 ± 8.7 years; range 0.01–40.0 years, accounting for 139,694 person-years), 22 (3.3%) of the NAFLD patients and 318 (4.9%) of the controls developed dementia (*p* = 0.07). The incidence rate of dementia was 1.7 per 1000 person-years (95% CI 1.1–2.6) in the NAFLD patients and 2.5 per 1000 person-years (95% CI 2.2–2.8) in the controls. The number of cases, incidence rates and HRs for dementia are shown in Table 2. Compared with the controls, we did not observe a significant association of NAFLD with dementia risk (HR 0.78; 95% CI 0.49–1.22). A similar result was obtained when we further adjusted for time-varying CVDs (adjusted HR [aHR] 0.77; 95% CI 0.48–1.21). Fig. 2 depicts the cumulative incidence of dementia in NAFLD compared with the matched controls.

**Table 2:** Number of cases, incidence rate (per 1000 person-years) and hazard ratios (crude and adjusted) for dementia.

	No.	Incidence rate (95% CI)	Crude HR (95% CI)∗	Adjusted HR (95% CI)†
Control	6,436	2.5 (2.2, 2.8)	Reference	Reference
NAFLD	656	1.7 (1.1, 2.6)	0.78 (0.49, 1.22)	0.77 (0.48, 1.21)
Fibrosis stage 0	163	0.6 (0.1, 2.3)	0.34 (0.08, 1.41)	0.33 (0.08, 1.37)
Fibrosis stage 1	256	1.7 (0.9, 3.3)	0.76 (0.37, 1.53)	0.82 (0.40, 1.64)
Fibrosis stage 2	149	2.6 (1.5, 6.1)	1.52 (0.70, 3.31)	1.44 (0.67, 3.12)
Fibrosis stage 3	58	0	n.a.	n.a.
Fibrosis stage 4	20	12.1 (3.9, 37.6)	1.73 (0.35, 8.53)	1.72 (0.35, 8.46)
Fibrosis stage 2–4	227	4.7 (2.5, 8.7)	1.04 (0.51, 2.12)	1.04 (0.52, 2.11)
Steatosis grade 1	228	2.4 (1.3, 4.4)	0.87 (0.43, 1.77)	0.88 (0.44, 1.79)
Steatosis grade 2	149	1.5 (0.5, 3.9)	0.58 (0.19, 1.49)	0.54 (0.19, 1.49)
Steatosis grade 3	203	2.0 (1.0, 4.0)	1.13 (0.53, 2.42)	1.12 (0.52, 2.39)
Lobular inflammation 0	52	1.9 (0.5, 7.8)	1.28 (0.28, 5.82)	1.31 (0.29, 5.98)
Lobular inflammation 1	245	1.7 (0.9, 3.3)	0.68 (0.34, 1.36)	0.67 (0.33, 1.34)
Lobular inflammation 2	220	1.8 (0.8, 3.9)	0.98 (0.44, 2.18)	1.00 (0.45, 2.25)
Lobular inflammation 3	62	3.9 (1.5, 10.5)	1.13 (0.33, 3.85)	1.09 (0.32, 3.74)
Ballooning 0	189	1.0 (0.4, 2.8)	0.49 (0.17, 1.36)	0.48 (0.17, 1.31)
Ballooning 1	207	2.4 (1.3, 4.5)	1.33 (0.66, 2.65)	1.37 (0.69, 2.74)
Ballooning 2	181	2.5 (1.3, 5.1)	0.80 (0.36, 1,77)	0.80 (0.36, 1.76)
Portal inflammation 0	254	1.3 (0.6, 2.7)	0.72 (0.33, 1.57)	0.72 (0.33, 1.57)
Portal inflammation 1	226	2.6 (1.5, 4.8)	0.96 (0.49, 1.91)	0.96 (0.49, 1.90)
Portal inflammation 2	99	2.6 (0.9, 6.8)	0.84 (0.29, 2.40)	0.84 (0.30, 2.41)
Non-NASH	194	1.0 (0.3, 2.7)	0.48 (0.17, 1.32)	0.46 (0.16, 1.28)
NASH	383	2.5 (1.5, 0.3)	1.04 (0.62, 1.75)	1.06 (0.63, 1.78)

na., not applicable; NAFLD, non-alcoholic fatty liver disease; NASH, non-alcoholic steatohepatitis.

∗ Conditional on matching variables: age, sex and municipality.

† Analysis adjusted for cardiovascular diseases as time-varying variable.

### Dementia risk factors in NAFLD patients

In patients with NAFLD, known risk factors for dementia from univariate Cox regression models are reported in Table 3. Factors associated with higher dementia risk (*p* <0.1) were older age (HR 1.16; 95% CI 1.10–1.23), female sex (HR 1.78; 95% CI 0.77–4.14), hypertension (HR 2.51; 95% CI 1.09–5.84) and CVDs (HR 2.27; 95% CI 1.00–5.31). These factors were further entered into a multi-adjusted model. The model, which included age, sex, hypertension, and CVDs, had a Harrell’s C index of 0.912 and served as the final crude model of conventional risk factors for dementia (Table S2).

**Table 3:** Hazard ratios with 95% confidence intervals (univariate and multivariate) for conventional risk factors for dementia in the histological cohort.

Parameter	All dementia	
**Univariate model**	**HR (95% CI)**	***P***
Age	1.16 (1.10, 1.23)	<0.001
Female sex	1.78 (0.77, 4.14)	0.100
Smoking status, ever	0.73 (0.30, 1.73)	0.471
Hypertension	2.51 (1.09, 5.84)	0.030
Cardiovascular diseases	2.27 (1.00, 5.31)	0.050
Obesity	0.95 (0.85, 1.06)	0.406
Type 2 diabetes	1.09 (0.25, 4.69)	0.909
**Multivariable model**	**Adjusted HR (95% CI)**∗	***P***
Age	1.17 (1.10, 1.23)	<0.001
Female sex	0.55 (0.21, 1.38)	0.213
Hypertension	1.95 (0.81, 4.71)	0.133
Cardiovascular diseases	1.34 (0.54, 3.33)	0.512
Harrell’s C-index†	0.912	

∗ The multivariable model adjusted for age, sex, hypertension, and time-varying cardiovascular diseases.

† Harrell’s C-index refers to the prediction ability of the multivariate model with the covariates age, sex, hypertension, and cardiovascular diseases.

Cox regression estimates and Harrell’s C-index for each histological parameter on the risk for dementia in patients with NAFLD are summarised in Table 4. Compared with those with fibrosis stage 0, a significant increase in dementia risk was observed in fibrosis stage 2 (aHR 4.91; 95% CI 1.02–23.4) and stage 4 (aHR 12.6; 95% CI 1.81–86.8) after adjusting for age, sex, hypertension, and CVDs. Because no dementia cases were diagnosed in patients with fibrosis stage 3, we combined those with fibrosis stage 2–4, from which we observed an increased risk of dementia (aHR 4.53; 95% CI 0.98–21.0). The model with age, sex, hypertension, CVDs, and fibrosis stage had a Harrell’s C-index of 0.938. Compared with the crude model of conventional risk factors (age, sex, hypertension, CVDs) with a Harrell’s C-index of 0.912, adding the presence of fibrosis to the model significantly increased the predictive power for dementia (LR test *p* = 0.008). The predictive ability also significantly increased, but to a lower extent than fibrosis stage, when individually adding NASH (C-index 0.927; LR test *p* = 0.001), ballooning stage (C-index 0.926; LR test *p* = 0.004), or portal inflammation stage (C-index 0.926; LR test *p* = 0.027) to the crude model, despite that no significant associations were found between these parameters and dementia risk. When adding different histological parameters simultaneously to the model, only fibrosis stage 4 was significantly associated with increased risk of dementia.

**Table 4:** Crude and multivariable hazard ratios and 95% confidence intervals for dementia risk and Harrell’s C-index by adding each histological parameter to a conventional risk model for dementia risk.

	Crude HR (95% CI)	Harrell’s C-index	Adjusted HR (95% CI)‡	Harrell’s C-index§	Likelihood-ratio test¶*p* value
NASH (yes)	2.95 (1.00–8.75)	0.600	2.93 (0.96–8.98)	0.927	0.001
Fibrosis (continuous)	1.96 (1.31–2.93)				
Fibrosis stage		0.766		0.938	0.019
Fibrosis stage 0	Reference				
Fibrosis stage 1	2.93 (0.63–13.6)		3.10 (0.65–14.6)		
Fibrosis stage 2	6.73 (1.42–31.8)∗		4.91 (1.02–23.4)∗		
Fibrosis stage 3	n.a.		n.a.		
Fibrosis stage 4	11.7 (1.71–79.7)∗		12.6 (1.81–86.8)∗		
Fibrosis stage 2-4	6.54 (1.43–29.6)∗		4.53 (0.98–21.0)†		
Steatosis		0.504		0.919	0.071
Steatosis grade 1	Reference				
Steatosis grade 2	0.71 (0.22–2.27)		0.73 (0.22–2.38)		
Steatosis grade 3	0.96 (0.38–2.43)		1.52 (0.57–4.05)		
Lobular inflammation		0.583		0.915	0.132
Lobular inflammation 0	Reference				
Lobular inflammation 1	0.89 (0.19–4.09)		0.87 (0.18–4.24)		
Lobular inflammation 2	1.26 (0.25–6.10)		1.28 (0.25–6.59)		
Lobular inflammation 3	2.80 (0.51–15.4)		2.09 (0.37–11.8)		
Ballooning stage		0.627		0.926	0.004
Ballooning 0	Reference				
Ballooning 1	2.58 (0.80–8.24)		2.97 (0.89–9.93)		
Ballooning 2	3.03 (0.91–10.2)		2.77 (0.82–9.51)		
Portal inflammation stage		0.615		0.926	0.027
Portal inflammation 0	Reference				
Portal inflammation 1	2.40 (0.92–6.22)		2.41 (0.92–6.36)		
Portal inflammation 2	2.76 (0.79–9.95)		1.90 (0.51–7.05)		

n.a., not applicable; NASH, non-alcoholic steatohepatitis.

∗ .*p* <0.05.

† *p* = 0.054.

‡ Model adjusted for age, sex, hypertension, and time-varying cardiovascular diseases.

§ Harrell’s C-index after adding an individual parameter to the conventional model of age, sex, hypertension, and cardiovascular diseases.

¶ Likelihood-ratio test compared with a conventional model of age, sex, hypertension, and cardiovascular diseases (compared with Harrell’s C-index = 0.912).

All the analyses were repeated for AD and non-AD dementia as the outcome (Tables S3 and S4). Compared with the reference population, no associations were observed for NAFLD with AD or non-AD. In the NAFLD cohort fibrosis stage, NASH, lobular inflammation, and ballooning, in general, were associated with a higher HR for non-AD after multi-adjustment, albeit insignificant. We did not observe a significant association between these histological parameters and AD.

When we repeated the analysis excluding people without information on histology (n = 10), the results were similar to the original analysis. The associations not materially altered after the exclusion of those <35 years old (Table S5). Smoking, T2DM, and BMI, added to the baseline model, yielded a C-index of 0.909 and adding the histological parameters to this full model still resulted in an increased risk prediction for dementia (C-index = 0.936 *vs.* C-index = 0.900, LR test *p* = 0.022) (Tables S2 and S6).

## Discussion

In this study of biopsy-proven NAFLD, no association was found between NAFLD and an increased risk of dementia, nor was any association found among subtypes (AD and non-AD) compared with a matched reference population. However, within the NAFLD cohort, data on histological parameters (especially fibrosis stage), increased the predictive power for dementia risk beyond the conventional risk model incorporating age, sex, hypertension, and CVDs.

To the best of our knowledge, very few studies have addressed the relationship between NAFLD and risk of incident dementia. One recent study found no increased risk of dementia in elderly patients with NAFLD, which corroborates with our results.^23^ Apart from this, limited clinical studies examining cognitive functioning in NAFLD patients have provided varying and conflicting results,[6], [7], [8]^,^^10^ discrepancies that might be as a result of methodological differences (*e.g.* use of different cognitive tests and the mean ages of the study population). One study from the UK with 224 participants with biopsy-proven NAFLD observed increased cognitive difficulties in NAFLD patients compared with healthy controls.^7^ Similar results were found among Korean and Turkish middle-aged adults, that NAFLD is independently associated with lower cognitive performance, regardless of CVD and cardiovascular risk factors.^6^^,^^8^ By contrast, the Framingham study, consisting of individuals with a mean age of 61 years, did not find an association between NAFLD and cognitive performance,^10^ which is consistent with our study demonstrating no increased risk for dementia in NAFLD. Although we do not have detailed information on risk factors for the controls, we cannot demonstrate that NAFLD *per se* is not independently associated with dementia risk. Given that NAFLD patients who underwent biopsy were routinely referred to hospital for check-ups where more CVD cases were being diagnosed (22.1% in NAFLD groups and 18.0% in matched controls), they might benefit more from better control of cardiovascular risk factors than the general population and therefore have less incident dementia cases (3.3% in NAFLD groups *vs.* 4.9% in matched controls). The association might be distorted by residual confounding as such. Thus, future studies that investigate whether NAFLD is independently associated with cognitive impairment or dementia are warranted.

Of note is the Framingham study showing that NAFLD patients with a high risk of advanced fibrosis (measured by a NAFLD fibrosis stage score) have lower cognitive function compared with those with low risk. In line with these results is our finding that advanced fibrosis stage might increase the risk of dementia after adjusting for a set of confounders. However, a small number of dementia cases were reported, and a dose–response pattern was partly found. Again, whether this suggests a causal relationship cannot be determined by the current study.

Accumulating evidence suggests that the fibrosis stage in NAFLD could be a risk factor for various diseases, including cerebrovascular diseases,^24^ carotid atherosclerosis,^25^ T2DM,^26^ and overall mortality.^14^ Given that fibrosis is also linked to brain white matter lesion and cognitive performance,^10^^,^^27^ Weinstein *et al*.^10^ suggested that cognitive impairment might be an additional consequence of NAFLD with fibrosis. This suggestion might be valid in individuals with advanced liver fibrosis, especially if it is comorbid with obesity. In such a condition the connective tissue in the liver will often accumulate under the circumstance of metabolic dysfunction and inflammation that, in turn, would have a detrimental effect on cognition.^22^^,^^28^ However, it is not evident whether liver fibrosis is independently associated with cognition, or whether it serves as a proxy for other mechanisms involving metabolic dysfunction or inflammation that promote brain alterations (*e.g.* small vessel diseases or macrovascular lesions) and eventually be responsible for cognitive impairment and the clinical manifestation of dementia.^29^ In fact, we found that histological markers, including hepatic fibrosis and NASH, significantly improve the predictive value for dementia risk beyond the conventional risk models of age, sex, hypertension, and CVDs, even though these risk factors already have a high predictive ability (Harrell’s C index = 0.912). These results might indicate that some of these histological markers *per se* might not associate with dementia risk independent of cardiovascular risk factors. Instead, they might share the metabolic milieu of insulin resistance,^30^ the secretion of adipokines,^31^ and oxidative stress^32^ and further facilitate and reinforce the deleterious effects of cardiometabolic risk factors on the brain. Additionally, the stronger association we found between histological markers and non-AD also indicates possible vascular pathology. This result is supported by previous work demonstrating that fibrosis severity was linked to executive function more prone to subclinical vascular injury,^33^ rather than other cognitive domains such as episodic memory.^10^

The main strength of our study is that all the NAFLD cases were diagnosed by liver biopsy, which is the gold standard for measuring liver fibrosis. All histological slides were of good quality so that the characteristics and severity of the NAFLD cases could be ascertained. Another strength is that we were able to distinguish histological parameters and assess their relationship with dementia separately. Additionally, this cohort is large and has the longest ever documented follow-up (mean 20 years) in biopsy-proven NAFLD.

There are significant limitations of this study. First, because of the nature of the study design, we are not able to obtain detailed information on clinical, biochemical, therapeutic, and other socioeconomic factors in the reference population apart from age, sex, and municipality. This limitation restricted our ability to examine whether NAFLD is an independent risk factor for dementia. Moreover, being that NAFLD is highly prevalent in the general population, there might be NAFLD cases in the matched controls, which could dilute the association between NAFLD and dementia. Nevertheless, we did not find a positive association between NAFLD and dementia risk. A second limitation concerns the possibility that the dementia cases were misclassified because the NPR only includes ICD diagnoses and it does not capture data from primary care. This possibility could explain the low prevalence of dementia in our study as opposed to a previous study showing a prevalence of dementia of about 11% (*vs.* 6.3% in our cohort) in older community-dwelling individuals aged >60 years.^34^ Furthermore, this misclassification might be differential because NAFLD patients were more likely to be followed in specialty care. Thus, the dementia cases were more likely to be detected compared with the controls and therefore the association between NAFLD and dementia might be overestimated. However, in this rather large cohort we did not observe an increased risk of dementia with NAFLD. A third limitation is that the results might not be generalised to patients who were diagnosed with NAFLD in primary care settings because those included in the study were diagnosed in specialty care and as such represent more severe cases of NAFLD. A fourth limitation is that residual confounding cannot be ruled out (*e.g.* insulin resistance, oxidative stress) in the biopsy cohort. Fifth, we cannot rule out that the dementia diagnosis might be misclassified as hepatic encephalopathy in patients with cirrhosis. Lastly, despite the extended follow-up, low statistical power owing to the small number of dementia cases limited our investigation to the temporal relation between NAFLD and dementia by restricting different follow-up intervals.

### Clinical implications

Although numerous dementia risk prediction models have been proposed,^13^^,^^35^ new risk factors and biomarkers were identified and incorporated into existing models to increase the predictive accuracy. Histological parameters might serve as such markers for dementia risk, which need to be corroborated in future studies. Clinicians could consider assessing dementia risk in patients with advanced fibrosis due to NAFLD, but not in the entire NAFLD population. Patients with NAFLD, especially those who are comorbid with metabolic syndrome and CVDs, should be closely monitored and treated for their concomitant cardiovascular risk factors to prevent dementing disorders, as these risk factors already account for a large part of an increased risk of dementia. In this regard, it would be useful if future clinical studies could assess whether any inflammation process associated with NAFLD is ameliorated in the brain if the CVDs are successfully treated, and whether the treatment targeting cardiovascular risk factors combined with fibrosis resolution significantly reduces the risk of dementia in patients with NAFLD.

## Conclusions

Patients with NAFLD are not at an increased risk of developing dementia than individuals in the general population. Conventional risk factors contribute to a higher risk of dementia and adding histological markers, especially fibrosis stage, can enhance the predictive power for incident dementia, indicating a shared metabolic milieu of aetiology towards dementia.

## Financial support

This research received no specific grant from any funding agency in the public, commercial, or not-for-profit sectors.

## Authors’ contributions

Study conception and design: YS, HH

Acquisition of data: HH, PN, ME

Statistical analysis: YS

Interpretation of data: All authors

Drafting of the manuscript: YS

Critical revision: All authors

Guarantor of the manuscript: HH

## Data availability

Data are subject to personal information protection regulations and are not publicly available. Sharing of anonymised data will be considered on a case-by-case basis on request.

## Conflict of interest

The authors declare no conflicts of interest that pertain to this work. Please refer to the accompanying ICMJE disclosure forms for further details.

## Supplementary data

Supplementary information.pdf

CTAT table.pdf
