# Supplementary material for: Non-alcoholic fatty liver disease does not increase dementia risk although histology data might improve risk prediction
Source: JHEP Rep. 2020 Dec 1;3(2):100218. doi: 10.1016/j.jhepr.2020.100218 (PMC7847958; doi:10.1016/j.jhepr.2020.100218)
Supplement: Supplementary information.pdf [file mmc1.pdf]

Non-alcoholic fatty liver disease does not increase dementia risk although histology data might improve risk prediction

Ying Shang, Patrik Nasr, Mattias Ekstedt, Linnea Widman, Per Stål, Rolf Hultcrantz, Stergios Kechagias, Hannes Hagström

Table of contents

Table S1..2

Table S2..4

Table S3..5

Table S4..6

Table S5..8

Table S6..9

Table S1. ICD codes for the diagnosis of dementia and cardiovascular diseases

**Dementia**	
**ICD-8**	
290.0	Senile dementia
290.1	Presenile dementia
**ICD-9**	
290.0	Senile dementia, uncomplicated
290.1	Presenile dementia
290.10	Presenile dementia, uncomplicated
290.11	Presenile dementia with delirium
290.12	Presenile dementia with delusional features
290.13	Presenile dementia with depressive features
290.2	Senile dementia with delusional or depressive features
290.20	Senile dementia with delusional features
290.21	Senile dementia with depressive features
290.3	Senile dementia with delirium
290.4	Arteriosclerotic dementia
290.40	Arteriosclerotic dementia, uncomplicated
290.41	Arteriosclerotic dementia with delirium
290.42	Arteriosclerotic dementia with delusional features
290.43	Arteriosclerotic dementia with depressive features
290.8	Other specified senile psychotic conditions
290.9	Unspecified senile psychotic condition
294.1	Dementia in conditions classified elsewhere
331	Other cerebral degenerations
331.0	Alzheimer's disease
331.1	Pick's disease
331.2	Senile degeneration of brain
**ICD-10**	
F00	Dementia in Alzheimer's disease
F01	Vascular dementia
F02	Dementia in other diseases classified elsewhere
F03	Unspecified dementia
G30.0	Alzheimer's disease with early onset
G30.1	Alzheimer's disease with late onset
G30.8	Other Alzheimer's disease
G30.9	Alzheimer's disease, unspecified
G31	Other degenerative diseases of nervous system, not elsewhere classified
G31.0	Circumscribed brain atrophy
G31.1	Senile degeneration of brain, not elsewhere classified
G31.2	Degeneration of nervous system due to alcohol
G31.8	Other specified degenerative diseases of nervous system
G31.9	Degenerative disease of nervous system, unspecified
**Cardiovascular diseases**	
**ICD-8**	

410	Acute myocardial infarction
411	Other acute and subacute forms of ischemic heart disease
412	Chronic ischemic heart disease
413	Angina pectoris
431	Cerebral hemorrhage
432	Occlusion of precerebral arteries
**ICD-9**	
410	Acute myocardial infarction
411	Other acute and subacute forms of ischemic heart disease
412	Old myocardial infarction
413	Angina pectoris
431	Intracerebral hemorrhage
433	Occlusion and stenosis of precerebral arteries
**ICD-10**	
I20	Angina pectoris
I21	Acute myocardial infarction
I22	Subsequent ST elevation (STEMI) and non-ST elevation (NSTEMI) myocardial infarction
I23	Certain current complications following ST elevation (STEMI) & non-ST elevation (NSTEMI) myocardial infarction (within 28 days)
I24	Other acute ischemic heart diseases
I61	Nontraumatic intracerebral hemorrhage
I63	Cerebral infarction
I64	Stroke, not specified as hemorrhage or infarction

Table S2- Harrell's C-index for different combinations of risk factors for dementia

Models	Harrel's C-index	Harrel's C-index after adding fibrosis stage	LR test P value
Age+Sex+Hypertension+CVD+type 2 diabetes+smoking+BMI	0.909	0.936	0.022
Age+Sex+Hypertension+CVD+ smoking+Obesity	0.910	0.937	0.008
Age+Sex+Hypertension+CVD+ BMI	0.911	0.937	0.038
Age+Sex+Hypertension+CVD+ smoking	0.909	0.921	0.043
Age+Sex+Hypertension+CVD+ type 2 diabetes	0.910	0.937	0.039
Age+Sex+Hypertension+CVD	0.912	0.938	0.008
Age+Sex+Hypertension	0.911	0.924	0.055
Age+Sex +CVD	0.905	0.918	0.019
Age+Hypertension+CVD	0.911	0.918	0.054
Age+Sex	0.907	0.919	0.039
Sex+Hypertension+CVD	0.662	0.756	0.003
Sex+ CVD	0.644	0745	0.002
Sex+Hypertension	0.637	0.737	0.004
Hypertension+CVD	0.619	0.741	0.003
Age	0.909	0.918	0.050
Sex	0.605	0.720	0.002
Hypertension	0.581	0.725	0.002
CVD	0.536	0.726	0.001
Smoking	0.508	0.725	0.001
Obesity	0.506	0.728	0.001
Type 2 diabetes	0.517	0.709	0.001

CVD, cardiovascular diseases; LR, Likelihood-ratio test

LR test compare the goodness of fit of two models with and without adding fibrosis stage.

Table S3- Hazard ratios for the risk of dementia subtype in relation to NAFLD compared with a matched reference population

	Alzheimer's disease		Non-Alzheimer's disease dementia	
	Crude HR (95% CI)[a]	Adjusted HR (95% CI)[b]	Crude HR (95% CI)[a]	Adjusted HR (95% CI)[b]
Control				
NADFL	0.83 (0.46, 1,48)	0.83 (0.46, 1.48)	0.78 (0.43, 1.40)	0.76 (0.43,1.36)
Histological sample				
Fibrosis stage 0	0.63 (0.14, 2.70)	0.63 (0.14, 2.68)	0.29 (0.04, 2.21)	0.30 (0.04, 2.22)
Fibrosis stage 1	0.79 (0.31, 2.01)	0.82 (0.32, 2.08)	0.72 (0.31, 1.71)	0.73 (0.31, 1.72)
Fibrosis stage 2	1.16 (0.39, 3.41)	1.13 (0.38, 3.31)	1.69 (0.64, 4.48)	1.57 (0.58, 4.25)
Fibrosis stage 3	-	-	-	-
Fibrosis stage 4	2.44 (0.45,12.9)	2.43 (0.45, 12.9)	1.51 (0.16, 13.7)	1.47 (0.16, 13.4)
Fibrosis stage 2-4	0.99 (0.42, 2.37)	0.99 (0.41, 2.36)	1.26 (0.52, 3.02)	1.14 (0.47, 2.75)
Steatosis grade 1	0.92 (0.38, 2.19)	0.92 (0.38, 2.19)	0.82 (0.32, 2.10)	0.84 (0.33, 2.17)
Steatosis grade 2	0.25 (0.03, 1.90)	0.26 (0.03, 1.89)	0.88 (0.31, 2.50)	0.85 (0.29, 2.45)
Steatosis grade 3	1.44 (0.59, 3.52)	1.44 (0.59, 3.52)	0.92 (0.32, 2.65)	0.87 (0.30, 2.52)
Lobular inflammation 0	1.97 (0.40, 9.57)	1.96 (0.40, 9.54)	0.96 (0.12, 7.85)	0.97 (0.12, 8.03)
Lobular inflammation 1	0.90 (0.40, 1.99)	0.90 (0.40, 2.00)	0.50 (0.17, 1.40)	0.47 (0.16, 1.33)
Lobular inflammation 2	0.74 (0.22, 2.47)	0.74 (0.22, 2.47)	1.47 (0.60, 3.58)	1.61 (0.66, 3.96)
Lobular inflammation 3	0.62 (0.08, 4.76)	0.60 (0.07, 4.74)	1.15 (0.25, 5.28)	1.00 (0.22, 4.64)
Ballooning 0	0.82 (0.29, 2.36)	0.82 (0.29, 2.37)	0.37 (0.09, 1.59)	0.34 (0.08, 1.43)
Ballooning 1	0.92 (0.32, 2.64)	0.92 (0.32, 2.63)	1.86 (0.83, 4.17)	2.01 (0.89, 4.54)
Ballooning 2	0.94 (0.37, 2.43)	0.94 (0.36, 2.43)	0.60 (0.18, 1.98)	0.58 (0.18, 1.94)
Portal inflammation 0	1.16 (0.48, 2.76)	0.15 (0.48, 2.75)	0.59 (0.21, 1.66)	0.58 (0.21, 1.64)
Portal inflammation 1	0.49 (0.15, 1.61)	0.49 (0.15, 1.61)	1.24 (0.58, 2.70)	1.25 (0.58, 2.71)
Portal inflammation 2	1.19 (0.40, 3.51)	1.18 (0.40, 3.51)	0.54 (0.07, 4.15)	0.52 (0.07, 3.97)
Non-NASH	0.80 (0.28, 2.30)	0.81 (0.28, 2.31)	0.36 (0.08, 1.51)	0.33 (0.07, 1.39)
NASH	0.94 (0.46, 1.89)	0.93 (0.46, 1.88)	1.18 (0.61, 2.27)	1.21 (0.63, 2.34)

NAFLD, nonalcoholic fatty liver disease; NASH, nonalcoholic steatohepatitis.

[a.] Analysis of the matching variables: age, sex and municipality.

[b.] Analysis adjusted for cardiovascular diseases as time-varying variable.

Table S4- Crude and multivariable hazard ratios for the risk of dementia subtype within the NAFLD cohort

	Alzheimer's disease		Non-Alzheimer's disease dementia	
	Crude HR (95% CI)	Adjusted HR (95% CI)[b]	Crude HR (95% CI)	Adjusted HR (95% CI)[b]
NASH (yes)	1.42 (0.43, 4.64)	1.54 (0.45, 5.28)	3.96 (0.88, 17.8)	3.88 (0.83, 18.0)
Fibrosis stage				
Fibrosis stage 0	Reference	Reference	Reference	Reference
Fibrosis stage 1	1.66 (0.32, 8.59)	2.07 (0.83, 12.3)	3.91 (0.47, 32.5)	4.30 (0.51, 36.5)
Fibrosis stage 2	3.29 (0.60, 18.1)	2.78 (0.49, 15.6)	8.53 (1.00, 73.4)[a]	5.98 (0.67, 51.9)
Fibrosis stage 3	NA	NA	NA	NA
Fibrosis stage 4	29.7 (3.79, 231.9)[a]	9.78 (1.19, 80.3)[a]	89.6 (7.27, 1104)[a]	22.3 (1.67, 299)[a]
Fibrosis stage 2-4	3.46 (0.69, 17.3)	2.75 (0.53, 14.3)	8.51 (1.04, 69.5)[a]	5.83 (0.69, 48.7)
Steatosis				
Steatosis grade 1	Reference	Reference	Reference	Reference
Steatosis grade 2	0.28 (0.03, 2.41)	0.35 (0.04, 2.99)	1.19 (0.33, 4.22)	1.21 (0.33, 4.41)
Steatosis grade 3	1.18 (0.38, 3.69)	2.24 (0.65, 7.81)	0.81 (0.22, 2.86)	1.21 (0.32, 4.57)
Lobular inflammation				
Lobular inflammation 0	Reference	Reference	Reference	Reference
Lobular inflammation 1	0.68 (0.14, 3.28)	0.62 (0.11, 3.22)	0.77 (0.09, 6.96)	0.68 (0.07, 6.41)
Lobular inflammation 2	0.50 (0.08, 3.06)	0.50 (0.08, 3.26)	2.25 (0.27, 18.8)	2.35 (0.27, 20.7)
Lobular inflammation 3	0.66 (0.06, 7.34)	0.53 (0.05, 6.08)	4.42 (0.45, 42.8)	3.54 (0.35, 35.6)
Ballooning stage				
Ballooning 0	Reference	Reference		
Ballooning 1	1.00 (0.25, 4.03)	1.24 (0.28, 5.31)	4.12 (0.87, 19.5)	4.78 (0.96, 23.8)
Ballooning 2	1.82 (0.48, 6.86)	1.82 (0.47, 7.08)	3.01 (0.56, 16.9)	2.74 (0.48, 15.5)
Portal inflammation stage				
Portal inflammation 0	Reference	Reference		

Portal inflammation 1	0.74 (0.19, 3.00)	0.84 (0.20, 3.47)	3.52 (1.08, 11.5)[a]	3.55 (1.06, 11.9)
Portal inflammation 2	3.15 (0.87, 11.4)	2.67 (0.65, 10.8)	1.25 (0.13, 11.3)	0.78 (0.08, 7.50)

NASH, nonalcoholic steatohepatitis. NA, not applicable.

[a.] $p<.05$

[b.] Model adjusted for age, sex, hypertension and time-varying cardiovascular diseases.

Table S5- Hazard ratios for the risk of dementia subtype in relation to NAFLD compared with a matched reference population, excluding individuals aged <35 years

	All dementia	Alzheimer's disease	Non-Alzheimer's disease
	HR (95% CI)[a]	HR (95% CI)[a]	HR (95% CI)[a]
Control	Reference	Reference	Reference
NAFLD	0.77 (0.49, 1.23)	0.83 (0.47, 1.49)	0.76 (0.43,1.37)
Fibrosis stage			
Fibrosis stage 0	0.33 (0.08, 1.37)	0.63 (0.14, 2.72)	0.30 (0.04, 2.23)
Fibrosis stage 1	0.77 (0.38, 1.54)	0.79 (0.31, 2.00)	0.73 (0.31, 1.72)
Fibrosis stage 2	1.54 (0.71, 3.36)	1.19 (0.40, 3.52)	1.58 (0.58, 4.25)
Fibrosis stage 3	NA	NA	NA
Fibrosis stage 4	1.72 (0.35, 8.46)	2.43 (0.46, 12.9)	1.47 (0.16, 13.4)
Fibrosis stage 2-4	1.10 (0.56, 2.16)	1.00 (0.42, 2.41)	1.14 (0.47, 2.76)
Steatosis			
Steatosis grade 1	0.93 (0.45, 1.89)	0.96 (0.40, 2.29)	0.88 (0.34, 2.28)
Steatosis grade 2	0.54 (0.19, 1.49)	0.30 (0.03, 1.89)	0.85 (0.29, 2.45)
Steatosis grade 3	1.14 (0.53, 2.43)	1.47 (0.60, 3.63)	0.87 (0.30, 2.52)
Lobular inflammation			
Lobular inflammation 0	1.31 (0.28, 5.98)	1.96 (0.40, 9.54)	0.98 (0.12, 8.03)
Lobular inflammation 1	0.67 (0.33, 1.35)	0.91 (0.40, 2.02)	0.48 (0.17, 1.33)
Lobular inflammation 2	1.00 (0.45, 2.24)	0.74 (0.22, 2.46)	1.61 (0.66, 3.95)
Lobular inflammation 3	1.09 (0.32, 3.74)	0.61 (0.07, 4.74)	1.21 (0.22, 4.65)
Ballooning stage			
Ballooning 0	0.47 (0.17, 1.33)	0.84 (0.29, 2.42)	0.34 (0.08, 1.43)
Ballooning 1	1.37 (0.68, 2.74)	0.92 (0.32, 2.63)	2.01 (0.89. 4.53)
Ballooning 2	0.80 (0.36, 1.76)	0.94 (0.36, 2.43)	0.59 (0.18, 1.95)
Portal inflammation stage			
Portal inflammation 0	0.72 (0.33, 1.59)	1.18 (0.49, 2.82)	0.58 (0.21, 1.64)
Portal inflammation 1	0.96 (0.49, 1.91)	0.49 (0.15, 1.61)	1.25 (0.58, 2.72)
Portal inflammation 2	0.85 (0.30, 2.41)	1.18 (0.40, 3.50)	0.52 (0.06, 3.97)
NAFLD	0.47 (0.17, 1.30)	0.82 (0.28, 2.36)	0.33 (0.08, 1.38)
NASH	1.06 (1.02, 1.70)	0.93 (0.68, 1.39)	1.21 (0.63, 2.33)

NAFLD, nonalcoholic fatty liver disease; NASH, nonalcoholic steatohepatitis; NA, not applicable.

[a.] Analysis adjusted for cardiovascular diseases as time-varying variable

Table S6. Multivariate adjusted hazard ratios of risk factors for dementia in the histological cohort

	All dementia	
Parameter	HR (95% CI)[a]	p
Age	1.17 (1.11, 1.24)	<0.001
Female sex	0.56 (0.21, 1.47)	0.242
Smoking status, ever	0.88 (0.50, 1.54)	0.661
Hypertension	1.95 (0.78, 4.90)	0.151
Cardiovascular diseases	1.45 (0.57, 3.67)	0.430
Obesity	1.00 (0.88, 1.14)	0.903
Type 2 diabetes	0.70 (0.14, 3.25)	0.661

[a.] The parameters are mutually adjusted in the multivariable model.
